# The effect of ultraviolet photofunctionalization of titanium instrumentation in lumbar fusion: a non-randomized controlled trial

**DOI:** 10.1186/s12891-019-2672-3

**Published:** 2019-06-18

**Authors:** Hiroyuki Tominaga, Kanehiro Matsuyama, Yukihiro Morimoto, Takuya Yamamoto, Setsuro Komiya, Yasuhiro Ishidou

**Affiliations:** 1 Department of Orthopaedic Surgery, Graduate School of Medical and Dental Sciences, Kagoshima University, 8-35-1 Sakuragaoka, Kagoshima, 890-8520 Japan; 2 Medical Joint Materials, Graduate School of Medical and Dental Sciences, Kagoshima University, 8-35-1 Sakuragaoka, Kagoshima, 890-8520 Japan; 3 USHIO INC, 1194, Sazuchi, Bessho-cho, Himeji, Hyogo 671-0224 Japan

**Keywords:** Ultraviolet (UV) photofunctionalization, Titanium, Instrumentation aging, Posterior lumbar interbody fusion

## Abstract

**Background:**

Titanium instrumentations are widely used in orthopedics; the metal bonds with bone in a process called osseointegration. Over time, hydrocarbons adhere to the instrumentation, which weakens the bone-binding ability. Ultraviolet photofunctionalization enhances the bone-binding ability of instrumentation by reducing hydrocarbons. The process has been proven effective in dentistry, but its effects in orthopedics are unverified. We aimed to determine the effect of ultraviolet photofunctionalization of titanium instrumentation used in lumbar fusion.

**Methods:**

This was a non-randomized controlled trial. We prospectively enrolled 13 patients who underwent lumbar fusion surgery. We inserted two pure titanium cages into each intervertebral space; one cage had undergone ultraviolet photofunctionalization, while the other was untreated. The degree of osteosclerosis around both cages was then compared by measuring the densities around the cages on imaging at 2, 3, 6, and 12 months postoperatively compared with 1 month postoperatively. The carbon attachment of the titanium cages was measured using X-ray photoelectron spectroscopy.

**Results:**

There was no significant difference between the degree of osteosclerosis (as assessed by the density) around the treated versus untreated cages at any timepoint. The ratio of carbon attachment of the titanium cages was only 20%, which was markedly less than the ratio of carbon attachment to titanium instrumentation previously reported in the dentistry field.

**Conclusions:**

The effect of ultraviolet photofunctionalization of titanium instrumentation in spine surgery is questionable at present. The biological aging of the titanium may be affected by differences in the manufacturing process of orthopedics instrumentation versus dentistry instrumentation.

**Trial registration:**

UMIN Clinical Trials Registry (Identifier: UMIN000014103; retrospectively registered on June 1, 2014).

## Background

Titanium instrumentation is widely used in the field of orthopedics because of its high osteoconductivity. The titanium instrumentations bond with bone in a process called osseointegration. However, hydrocarbon attaches to the instrumentation over time, causing the bone-seeking aptitude of the titanium instrumentation to decrease [1, 2].

Ultraviolet (UV) photofunctionalization is a technique that has been recently developed to improve the biological aging of titanium instrumentation in the field of dentistry [3–9]. Aita et al. used UV photofunctionalization technology to create a super-hydrophilic state to remove hydrocarbon from the titanium surface, which substantially increased the migration, adhesion, survival, and growth of osteogenic cells, and increased the onset of instrumentation fixation [1]. However, the utility of UV photofunctionalization in the orthopedic field is unknown. The present study aimed to determine the effect of UV functionalization of titanium instrumentation in spine surgery.

## Methods

Our hypothesis was that UV photofunctionalization of titanium instrumentation would decrease the amount of carbon that built up on the instrumentation surface over time, and would therefore increase the strength of the bone–instrumentation bond.

We prospectively enrolled 13 patients who underwent lumbar fusion from January 2014 to December 2015. The average age of the patients is 69 years old (from 55 to 82). We used the Prospace® intervertebral cage (B-Braun Company, Melsungen, Germany), which has a surface made of pure titanium that covers a Ti-6Al-4 V alloy. The cages are coated with a layer of fine titanium powder applied in a plasmaspray process under vacuum. The pore sizes range from 50 to 200 μm with a microporosity of 37.3%. We inserted two cages into each intervertebral space, one with and one without UV photofunctionalization, and subsequently compared the degree of osteosclerosis around both cages. The side that received the UV spacer was randomly decided. UV photofunctionalization of the cages was performed for 15 min using a low-pressure mercury lamp (TheraBeam Affinity®, USHIO INC., Himeji, Japan). Using the lamp, samples were exposed to UV light from both sides for 15 min. The main wavelength of the light to which the specimen was exposed was 254 nm, and the illuminance at the surface of the sample was 9.5 mW/cm^2^. Spine surgeons who were instructors accredited by the Japanese Society for Spine Surgery and Related Research performed the lumbar fusion surgeries. We excluded cases in which the disc wedging was more than 5°. There was no laterality of the autograft mass in the disc space.

**Fig. 1:**
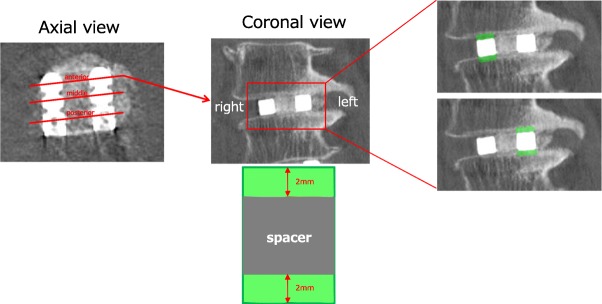
CT images showing the method used to measure bone sclerosis. The red lines in the left image represent the anterior, middle, and posterior regions of the intervertebral spacer on an axial CT image. We measured the mean range of image in the 2 mm above and below each of these three regions on coronal CT images (middle and right images). Density range: 0 (black)–255 (white)

The density around the titanium instrumentation with versus without UV photofunctionalization was assessed to evaluate the degree of osseointegration. We detected the ossification around the cage using Win ROOF 2013 imaging analysis software (Mitani Co., Fukui, Japan). On computed tomography (CT) coronal images, the mean range of image (ROI) was measured in the 2 mm directly above and below the anterior, intermediate, and posterior regions of each intervertebral spacer (Fig. 1). We measured the ROI around the cage at 1, 2, 3, 6, and 12 months postoperatively. The difference in ROI over time was measured compared with the value at 1 month postoperatively.

The carbon attachment of the titanium cage was measured using the X-ray photoelectron spectroscopy (XPS) method, which analyzes the constituent element of the sample and the electronic state [10]. By measuring the energy distribution of photoelectrons generated after irradiation with X-rays on the cage surface, we were able to analyze the constituent elements and their electronic states.

The degree of osteosclerosis around the titanium implants were compared using the Mann–Whitney U test. *P* < 0.05 was considered statistically significant.

The software used for statistical analyses was BellCurve for Excel (Social Survey Research Information Co., Ltd. Tokyo, Japan), which is add-in Excel software for statistical evaluation.

## Results

**Fig. 2:**
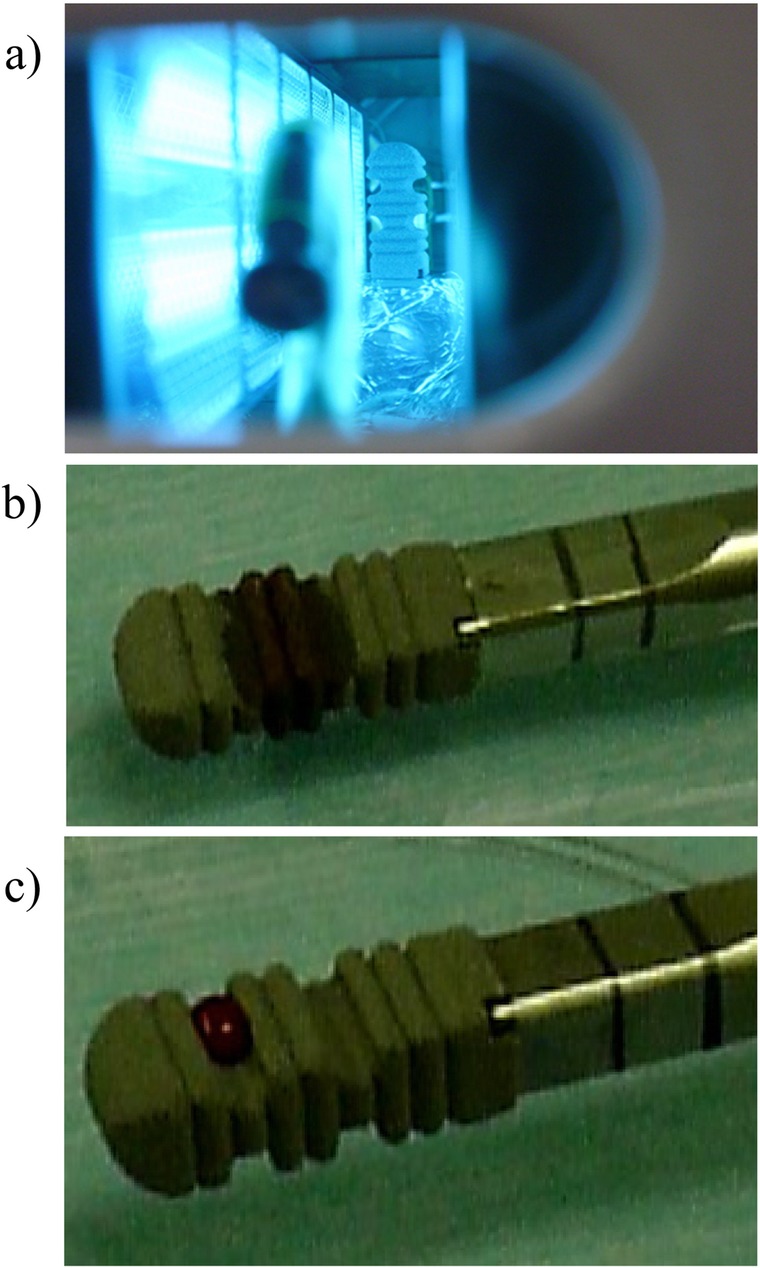
The effect of ultraviolet (UV) photofunctionalization. a. Photograph of an intervertebral spacer undergoing UV photofunctionalization. b. The intervertebral spacer became hydrophilic after UV photofunctionalization. Blood was absorbed into the cage that underwent UV photofunctionalization, immediately after the drop was applied. c. The intervertebral spacer remained hydrophobic without UV photofunctionalization. Absorption of the blood drop into the cage without UV photofunctionalization was delayed

**Fig. 3:**
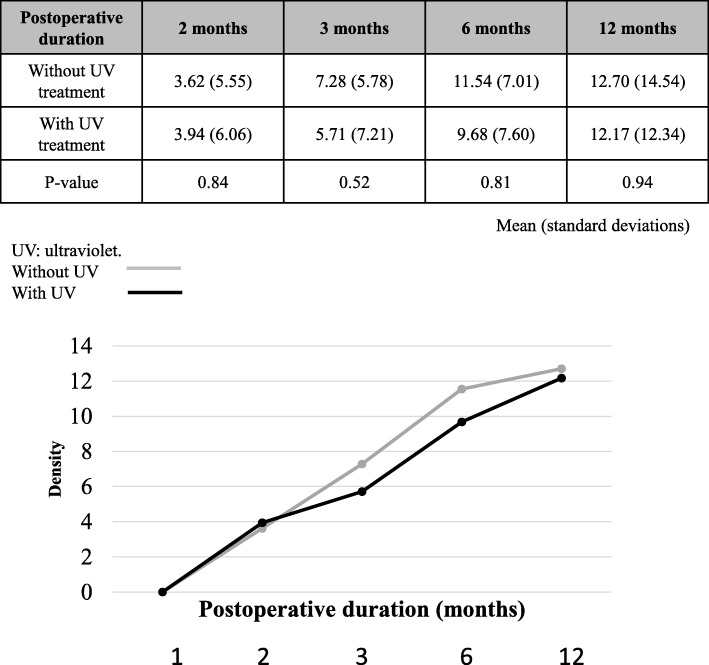
Changes in density over time. Postoperative duration indicates the length of time after lumbar fusion surgery performed using titanium instrumentation either without UV treatment or with UV treatment. There were no significant differences in the density of the titanium implants that were UV-treated versus those that were not UV-treated at any timepoint (Mann-Whitney U test). (2 months: *P* = 0.84, 3 months: *P* = 0.52, 6 months: *P* = 0.81, 12 months: *P* = 0.94)

The intervertebral spacers became hydrophilic after UV photofunctionalization (Fig. 2). The osteosclerosis around the instrumentation (as indicated by increases in density) was reinforced over time in all 13 patients (Fig. 3). However, there was no significant difference in the density around UV-treated versus non-UV-treated instrumentation at any timepoint compared with 1 month postoperatively (Fig. 3).

**Fig. 4:**
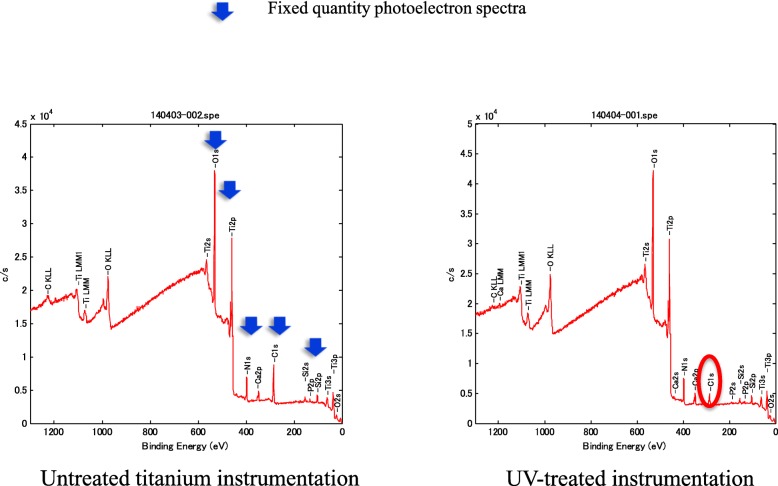
Composition of the surface elemental analysis by X-ray photoelectron spectroscopy. By measuring the energy distribution of photoelectrons generated after irradiation with X-rays on the sample surface, we were able to analyze the constituent elements and electronic states of our instrumentation samples. The actual titanium instrumentation used in lumbar fusion surgery 1 year after production is shown. We used PROSPACE® intervertebral cages (B-Braun Company, Melsungen, Germany) in all patients in this study. The surface of the PROSPACE® is pure titanium, which covers a Ti-6Al-4 V alloy. Actual titanium instrumentation that underwent ultraviolet photofunctionalization had a decreased amount of carbon attached to it. Vertical axis: photoelectron speed (C/S: counts / sec). Horizontal axis: binding energy (eV: electronvolt)

The XPS analysis of the surface elements of the instrumentation showed that the amount of carbon attached to the titanium spacer was decreased by UV photofunctionalization (Fig. 4, Table 1). The ratio of carbon attachment to the titanium cages was only 20%, even at 1 year after production (Table 1).

**Table 1:** X-ray photoelectron spectroscopy results

Sample	Element	Pre-UV	Post-UV	Change ratio (%)
a) Test piece of pure titanium evaluated 3 months after production				
1	O1s	48	58	+20.8
	**C1s**	**22**	**7**	**-68.2**
	Ti2p	5	6	+20.0
	N1s	11	11	0
	Si2p	13	15	+15.4
	P2p	0	0	0
	Ca2p	0	0	0
2	O1s	47	56	+19.1
	**C1s**	**25**	**9**	**-64.0**
	Ti2p	4	5	+25.0
	N1s	9	12	+33.3
	Si2p	13	15	+15.4
	P2p	0	0	0
	Ca2p	0	0	0
b) Test piece of the Ti-6Al-4V alloy evaluated 3 months after production				
1	O1s	49	62	+26.5
	**C1s**	**23**	**5**	**-78.3**
	Ti2p	3	4	+33.3
	N1s	7	8	+14.3
	Si2p	16	19	+18.8
	P2p	0	0	0
	Ca2p	0	0	0
2	O1s	47	59	+25.5
	**C1s**	**25**	**7**	**-72.0**
	Ti2p	2	3	+50.0
	N1s	10	10	0
	Si2p	14	18	+28.6
	P2p	0	0	0
	Ca2p	0	0	0
c) Actual implants (PROSPACE®) used in lumbar fusion surgery, produced 1 year prior to surgery				
1	O1s	40.8	50.4	+23.5
	**C1s**	**24.8**	**9.2**	**-62.9**
	Ti2p	19.3	22.5	+16.6
	N1s	10.1	11.5	+13.9
	Si2p	3.2	4.1	+28.1
	P2p	0.6	0.8	+33.3
	Ca2p	1.1	1.4	+27.3

*C1s* Energy peak position of carbon

*UV* Ultraviolet photofunctionalization

Carbon concentration = (The number of counts of carbon detected by X-ray photoelectron spectroscopy)/(The number of all elements detected by X-ray photoelectron spectroscopy)* 100

Boldface: The change ratio of carbon

## Discussion

To our knowledge, our study is the first to report the effects of UV photofunctionalization technology in orthopedics. UV photofunctionalization technology has been used to create a super-hydrophilic state to remove hydrocarbon from the titanium surface of dental instrumentation, which markedly increases the migration, adhesion, survival, and growth of osseous system cells, and increases the onset of implant fixation [1]. A previous animal experiment in the field of dentistry showed that UV photofunctionalization increased the bone–implant contact rate to approximately 100%, and increased the bond strength of bone–implant fixation by 2.5 times to more than three times that of the bond acquired using an implant that had not undergone UV treatment [1]. Furthermore, the percentage of carbon on the titanium surface of dentistry instrumentation just after production was approximately 10%, but this reached approximately 60% at the surface after 4 weeks [2]. We confirmed that UV photofunctionalization was effective in creating a hydrophilic state in the titanium instrumentation used in orthopedic surgery. However, in the present study, the osteosclerosis region around the instrumentation did not differ between cages that underwent UV photofunctionalization versus untreated cages. The carbon ratio of the instrumentation surface was decreased by UV photofunctionalization, but the carbon ratio of the normal instrumentation surface was about 20–30% at 1 year after implantation, which is lower than that reported in dentistry. The production of instrumentation is not the same in the field of orthopedics as in dentistry. The surfaces of instrumentation used in dentistry are treated with sulfuric acid, as treatment of the instrumentation surfaces with strong acid reportedly strengthens the binding between instrumentation and bone [11]. Moreover, fluorine coating of the surfaces reportedly strengthens bone conduction and calcification [12]. These differences in the production of instrumentation in the field of orthopedic surgery versus dentistry is one potential reason why UV photofunctionalization was not effective at increasing bone–implant fixation in the present study. We suggest that the manufacturing process of instrumentation in orthopedics makes it difficult to process the biological aging.

In clinical practice, titanium instrumentation treated by UV photofunctionalization reportedly prevented the growth of oral bacteria and biofilms [13, 14]. Infection is also an important issue affecting the clinical success of artificial joint instrumentation [15]; therefore, UV photofunctionalization may confer a great advantage in increasing resistance to infection following artificial joint replacement.

This study had several limitations. First, we used only one type of instrumentation, and did not investigate all instrumentation types used in orthopedics. Second, there were only a few cases evaluated. Finally, the test pieces and products were not used just after production.

## Conclusion

In conclusion, the effect of UV photofunctionalization of titanium instrumentation used in spine surgery is questionable at present; this may be due to the differences in the manufacturing process of orthopedics instrumentation versus dentistry instrumentation.

## Data Availability

The data set supporting the conclusion of this article is available on request to the corresponding author.

## Abbreviations

CT: Computed tomography
ROI: Range of image
UV: Ultraviolet
XPS: The X-ray photoelectron spectroscopy
